# Fractal motor activity during wakefulness and sleep: a window into depression recency and symptom recurrence

**DOI:** 10.1017/S0033291724002769

**Published:** 2024-11

**Authors:** O. Minaeva, H. Riese, S. H. Booij, F. Lamers, E. J. Giltay, F. A. J. L. Scheer, K. Hu

**Affiliations:** 1 Department of Psychiatry, University of Groningen, University Medical Center Groningen, Interdisciplinary Center Psychopathology and Emotion regulation, Groningen, The Netherlands; 2 Lentis, Center for Integrative Psychiatry, Groningen, The Netherlands; 3 Department of Psychiatry, Amsterdam UMC location Vrije Universiteit Amsterdam, Amsterdam, The Netherlands; 4 Amsterdam Public Health, Mental Health Program, Amsterdam, The Netherlands; 5 Department of Psychiatry, Leiden University Medical Center, Leiden, The Netherlands; 6 Division of Sleep Medicine, Harvard Medical School, Boston, MA, USA; 7 Medical Chronobiology Program, Division of Sleep and Circadian Disorders, Brigham and Women's Hospital, Boston, MA, USA; 8 Medical Biodynamics Program, Division of Sleep and Circadian Disorders, Brigham and Women's Hospital, Boston, MA, USA

**Keywords:** actigraphy, depression, fractal patterns, mood disorder, scale invariance, sleep–wake rhythm

## Abstract

**Background:**

Motor activity fluctuations in healthy adults exhibit fractal patterns characterized by consistent temporal correlations across wide-ranging time scales. However, these patterns are disrupted by aging and psychiatric conditions. This study aims to investigate how fractal patterns vary across the sleep–wake cycle, differ based on individuals' recency of depression diagnosis, and change before and after a depressive episode.

**Methods:**

Using actigraphy from two cohorts (*n* = 378), we examined fractal motor activity patterns both between individuals without depression and with varying recencies of depression and within individuals before and after depressive symptom recurrence. To evaluate fractal patterns, we quantified temporal correlations in motor activity fluctuations across different time scales using a scaling exponent, *α*. Linear mixed models were utilized to assess the influence of the sleep–wake cycle, (recency of) depression, and their interaction on *α*.

**Results:**

Fractal activity patterns in all individuals varied across the sleep–wake cycle, showing stronger temporal correlations during wakefulness (larger *α* = 1.035 ± 0.003) and more random activity fluctuations during sleep (smaller *α* = 0.784 ± 0.004, *p* < 0.001). This sleep–wake difference was reduced in recently depressed individuals (1–6 months), leading to larger *α* during sleep (0.836 ± 0.017), compared to currently depressed (0.781 ± 0.018, *p* = 0.006), remitted (0.776 ± 0.014, *p* < 0.001), and never-depressed individuals (0.773 ± 0.016, *p* < 0.001). Moreover, remitted individuals who experienced depressive symptom recurrence during antidepressant tapering exhibited a larger *α* during sleep after the symptom onset as compared to before (after: *α* = 0.703 ± 0.022; before: *α* = 0.680 ± 0.022; *p* < 0.001).

**Conclusions:**

These findings suggest a link between fractal motor activity patterns during sleep and depressive symptom recurrence in remitted individuals and those with recent depression.

## Introduction

Depressive disorders are increasingly prevalent mental health conditions that are linked to reduced quality of life and increased morbidity and mortality (World Health Organization, 2019). As a leading cause of disability, depression increases the risks for cardiovascular disease, hypertension, diabetes, dementia, and other chronic conditions (Byers & Yaffe, 2011; Kessler et al., 2009). However, timely detection and treatment of depression remain challenging, especially in primary care settings where many individuals initially present with depressive symptoms (van Weel, van Weel-Baumgarten, & van Rijswijk, 2009), which often leads to untreated cases and prolonged suffering for patients (Fernández et al., 2010). Thus, it is urgent to improve the detection of depression in ambulatory settings (Smagula, 2016).

One barrier to timely intervention is the absence of objective and scalable measures for assessing depression and its progression reliably (Campbell et al., 2018). Such measures would be of great value not only for detecting a depressive episode but also for evaluating the response to treatment. Recent advances in wearable devices, such as actigraphy, have triggered the discussion about their utility in clinical research and practice (Inan et al., 2020). However, effectively translating ambulatory recordings into meaningful clinical insights remains a complex task due to the lack of robust algorithms and target variables resilient to external influences (e.g. scheduled daily activities and environmental conditions) (Rykov, Thach, Bojic, Christopoulos, & Car, 2021).

Fractal physiology – an emerging field of medicine (Li et al., 2017b) – offers potential approaches to addressing this challenge. Outputs from healthy biological systems, including motor activity, display fractal temporal fluctuations with similar temporal structures across different time scales (Hu, Scheer, Ivanov, Buijs, & Shea, 2007). Independent of scheduled daily events and environmental conditions, these fractal fluctuations, characterized by a balance between randomness and excessive regularity, are believed to reflect the system's adaptability and integrity (Hu et al., 2004). Supporting this concept in fractal physiology, fractal patterns were found to be altered with aging and under pathological conditions, in which systems become less adaptive to perturbations and more vulnerable to catastrophic events (Pittman-Polletta, Scheer, Butler, Shea, & Hu, 2013). For instance, fractal patterns in human motor activity are degraded with aging, and the degradation is accelerated during the progression of Alzheimer's disease (Li et al., 2019b). Additionally, degraded fractal motor activity fluctuations are linked to the neuropathology of Alzheimer's disease (Gao et al., 2021) and precede many adverse health outcomes such as dementia, disability, frailty, and mortality (Li et al., 2019a).

The relationship between fractal activity patterns and depression remains unclear. A pilot study reported altered fractal activity patterns between 10 AM and 4 PM in 10 individuals with major depression as compared to 10 matched controls (Aybek et al., 2012). Replication and further validation of the results require studies of larger samples. In addition, no studies have examined how fractal activity patterns vary in individuals with depression across the sleep–wake cycle, and change across various recencies of depression (i.e. current, recent, or remitted depression) or during antidepressant tapering. Addressing these questions might help determine the potential of using fractal activity patterns as a diagnostic measure or early warning sign for a depression episode (Cramer et al., 2016).

To address these gaps, we studied motor activity recordings in two cohorts: (1) the Netherlands Study of Depression and Anxiety (NESDA) (Penninx et al., 2021) and (2) The TRANSitions In Depression (TRANS-ID) Tapering study (Kunkels et al., 2023; Smit et al., 2020). NESDA provides a large sample with different recencies of a depression diagnosis (i.e. current, recent, or remitted depression) and a group of never-depressed individuals, which is suitable for between-subject comparisons. TRANS-ID provides ultra-long motor activity recordings of remitted individuals when tapering their antidepressant medication, which allows for examining within-subject changes during the tapering. Using the two databases, we examined fractal activity patterns during sleep and wakeful periods and tested two hypotheses: (1) fractal patterns in motor activity are disrupted in individuals with (a history of) depression, especially with more recent depression diagnoses or symptom recurrence (NESDA: between-subject approach, TRANS-ID: within-subject approach), and (2) fractal activity patterns predict subsequent development of depressive symptoms in remitted individuals (TRANS-ID: between-subject approach).

## Methods

### Participants

We studied 378 individuals in the NESDA and TRANS-ID cohorts.

NESDA is an ongoing multisite longitudinal cohort study aimed at comparing individuals with varying recency of depression diagnoses. It included 2981 participants (18–65 years old) with or without depressive and anxiety disorders who were recruited from the community, primary care, and specialized mental healthcare settings (Penninx et al., 2021). A subset of participants from the Ecological Momentary Assessment & Actigraphy sub-study (*n* = 384) who underwent actigraphy assessment at the 9-year follow-up wave of NESDA (Difrancesco et al., 2019) was used in this study. The inclusion process of the current study is provided in Supplementary Material 1 (Fig. S1a). The final sample (*n* = 327) consisted of four groups: 43 participants with a depression diagnosis within the past month (‘current depression’), 47 with a depression diagnosis within the past 6 months but not within the past 1 month (‘recent depression’), 151 with a depression history but no diagnosis within the last 6 months (‘remitted depression’), and 86 participants who never had a depression diagnosis (‘never depressed’). These groups of participants were used to determine the differences in fractal activity patterns between individuals with different recencies of depression and never-depressed participants (hypothesis 1).

TRANS-ID sample included 69 remitted individuals with a past depressive episode who tapered their antidepressant medication during the study timeframe and were, therefore, at high risk of developing depressive symptoms (Geddes et al., 2003). The inclusion process of the current study is shown in Supplementary Material 1 (Fig. S1b). We included 50 participants with up to 4 months of actigraphy recording (Kunkels et al., 2023) which included 1 month before the end of tapering and 3 months after that (Smit et al., 2020). Such a design was chosen to capture the time window when any depressive symptoms were most likely to return (especially in the first month after tapering) (Smit, Snippe, Bringmann, Hoenders, & Wichers, 2022).

### Depression diagnosis and depressive symptom evaluation

In NESDA, depression diagnosis (MDD and/or dysthymia) was based on DSM-IV criteria (Bell, 1994) and assessed with the Composite International Diagnostic Interview (CIDI), version 2.1 (Wittchen, 1994). During a regular NESDA interview (scheduled no more than 31 days before the actigraphy assessment), the CIDI assessment was conducted to determine participants' previous and current depression status. Depression severity was assessed with the self-reported 30-item Inventory of Depressive Symptomatology (IDS) (Rush et al., 1996): a score of 0–13 for no to mild, 14–25 for mild, 26–38 for moderate, and 39 or higher for (very) severe depressive symptoms.

In TRANS-ID, depressive symptoms were assessed with the Symptom Checklist (SCL)-90 depression subscale (Derogatis & Unger, 2010). Participants filled out the questionnaires on their smartphones weekly for 6 months (~26 assessments per participant). Measurements started simultaneously with the actigraphy assessment and continued for 2 months after the end of the 4-month actigraphy assessment. Depressive symptom recurrence was identified using a combination of quantitative and qualitative criteria (Smit et al., 2022). Quantitatively, recurrence was fulfilled if there was a consistent increase in the Symptom Checklist-90 (SCL-90) depression subscale score over three consecutive weeks, with an increase of 8.5 points or more from the average score of the initial 2 weeks. The increase of at least 8.5 points on the SCL-90 was based on the Reliable Change Index (RCI), indicating the significant change at *α* = 0.05, calculated using the standard error of measurement (S.E.M. = 3.55 or 3.50) (Arrindell & Ettema, 2003; Jacobson & Truax, 1991). Qualitative criteria were assessed through participant self-reports and researcher evaluations, using data from questionnaires, phone calls, and interviews, which were independently rated by two psychologists and one psychiatrist based on predefined guidelines. Both quantitative and qualitative criteria needed to be met to confirm the incidence of ‘depressive symptom recurrence’. Upon identifying a recurrence period, the timeline was segmented into three phases: before, during, and after the onset. The period preceding the onset of recurrence was labeled ‘before the onset’, the initial week of recurrence as ‘during the onset’, and the subsequent time window as ‘after the onset’. For individuals without depressive symptom recurrence, the entire duration of actigraphy monitoring was categorized as ‘before the onset’.

### Actigraphy assessment and preprocessing

The motor activity of NESDA participants was monitored continuously for 14 days using the wrist-worn GENEActiv accelerometer (Activinsights Ltd., Kimbolton, UK) (Pavey, Gomersall, Clark, & Brown, 2016). This device captured three-axis acceleration data sampled at 30 Hz, producing activity counts (AC) in 1 min epochs. Participants were instructed to wear the accelerometer continuously throughout the day and night, removing it only when safety concerns arose, such as during activities like sauna sessions or contact sports. For the NESDA sample, the entire 14-day actigraphy monitoring period was used for analysis.

Raw data preprocessing was performed using Matlab version R2021B, with detailed steps outlined in Supplementary Material 2. Calibration error was checked against local gravity, and abnormal values and non-wear periods were identified and addressed. Objective motor activity measures were then extracted, and AC were calculated using a dedicated Matlab program (Supplementary Material 3). Missing or invalid data, such as suspected non-wear periods, were marked as ‘gaps’ and excluded from subsequent analyses. Only respondents with valid actigraphy data for a minimum of five consecutive days were included (*n* = 327). Missing data ranged from 0 to 76.2 h per person (0–22.7%), with the majority of participants experiencing minimal missing data.

The motor activity of TRANS-ID participants was monitored continuously for 4 months using the MotionWatch 8 (CamNTech, UK). The device sampled tri-axis acceleration at 3–11 Hz and generated AC with an epoch length of 1 min, which sampled tri-axis acceleration at 3–11 Hz and generated AC with 1 min epochs. Similar to NESDA participants, TRANS-ID participants were instructed to wear the device continuously throughout the day and night, with exceptions for safety-related situations (Kunkels et al., 2023). In the TRANS-ID study, the full 4-month monitoring period was used to capture long-term fluctuations and patterns in motor activity. Thus, for participants who experienced recurrence (*n* = 31), data before, during, and after the onset of depressive symptoms were included. For the comparison between individuals with and without a recurrence (*n* = 50), data before the onset of depressive symptoms were used for those participants with recurrence, while the entire actigraphy recording was analyzed for those without recurrence.

Preprocessing of TRANS-ID data was conducted using the native MotionWare software (v. 1.2.28). Due to battery limitations, the MotionWatch 8 devices were replaced midway through monitoring for each participant, and the resulting actigraphy files were merged for analysis. Missing data periods were marked as ‘gaps’ and excluded from subsequent analyses, with no data imputation performed. Only respondents with valid actigraphy data for at least seven uninterrupted days were included (*n* = 50). Missing data varied from 0 to 675.7 h per person (0–24.1%; median: 12.2 h or 0.4%; IQR: 110.5 h or 3.8%).

### Sleep–wake schedule

Times of going to bed and waking up for each day were determined using actigraphy data (NESDA) or combining actigraphy data and even markers (TRANS-ID). Based on the derived sleep/wake schedules, each of the six non-overlapping 4 h segments in each day was assigned to one of four states: sleep, sleep–wake transition, wake, and wake–sleep transition.

This was accomplished through an algorithm developed by Van Hees for sleep detection (Van Hees et al., 2015). To validate these estimates, we compared them to the habitual sleep–wake schedules reported on working and weekend days in the Munich Chronotype Questionnaire (MCTQ) (Roenneberg, Wirz-Justice, & Merrow, 2003). Adequate agreement was defined as being within a ±1 h window. Any sleep estimates lacking this agreement were excluded from the analysis, accounting for 20.8% of nights in total.

For TRANS-ID participants, we determined daily sleep–wake patterns similarly, utilizing both actigraphy recordings and event markers. Participants were instructed to use an event marker to indicate when they attempted to fall asleep and when they woke up. These markers, along with actigraphy data, were analyzed using MotionWare software version 1.3.17.

### Assessment of fractal activity patterns

To assess fractal activity patterns and their alterations, we performed detrended fluctuation analysis (DFA) that quantifies the temporal correlation property in motor activity fluctuations across different time scales (Hu, Ivanov, Chen, Carpena, & Stanley, 2001). The DFA algorithm is implemented in the ezActi software (Li, 2023), which was specifically designed for analyzing actigraphy data. The DFA algorithm includes five key steps: (i) integrating the AC after removing the global mean; (ii) dividing the integrated signal into non-overlapping windows of the same size with a given time scale (*n*); (iii) removing trends within each window using second-order polynomial fitting; (iv) calculating the root mean square of residuals to obtain the fluctuation amplitude *F*(*n*) at the time scale *n*. The four steps were repeated across different time scales; and (v) obtaining a scaling exponent, *α*, within a selected range of time scales by fitting the fluctuation amplitude function with a power-law function: *F*(*n*)~*n^α^*. *α* estimates the temporal correlation in the activity fluctuations within the timescale region: *α* = 0.5 indicates white noise (total randomness), *α* > 0.5 indicates positive correlations, where large AC are more likely followed by large values and vice versa, *α* close to 1.5 indicates excessive correlations or ‘too regular’, and *α* around 1.0 indicates the most complex fluctuations (i.e. a balance between randomness and excessive regularity). This study focused on *α* within two distinct time-scale regions: <90 min (*α*1) and 2–8 h (*α*2) that showed differential changes with aging and diseases (Hu, Van Someren, Shea, & Scheer, 2009; Li et al., 2019b).

To examine the overall fractal regulation across both timescale regions, weekly *α*1 and *α*2 were obtained for each participant by performing the DFA using the data of the whole week. Additionally, Δ*α*, representing the difference between *α*1 and *α*2, was calculated to assess the overall breakdown of fractal activity regulation. A larger Δ*α* indicates more disrupted fractal regulation, which has been associated with greater neuronal loss in the central circadian clock (Hu, Harper, Shea, Stopa, & Scheer, 2013).

To explore the variation of fractal activity patterns across the sleep–wake cycle, *α*1 was obtained using a 4 h sliding window, resulting in six *α*1 values per day. To ensure data quality, we checked the goodness of fit (i.e. *R*^2^ of the power-law fit of DFA-derived fluctuation amplitude function *F*(*n*)) and excluded those 1-week exponents with fitting goodness less than 0.9 and those 4 h exponents with fitting goodness less than 0.8 for wakefulness and less than 0.4 for sleep. In the NESDA dataset, 3 out of 1292 1-week exponents were excluded based on this criterion, and 2 out of 1549 1-week exponents were excluded from the TRANS-ID dataset. Additionally, for the 4 h exponents, 305 out of 27 587 were removed from the NESDA dataset, and 323 out of 32 344 were removed from the TRANS-ID dataset. Additionally, we calculated the standard deviation of *α*1 within each day as another measure of sleep–wake variation (intra-daily variation). Consequently, for each participant, five types of fractal activity metrics were assessed: weekly *α*1, *α*2, and Δ*α*, 4 h *α*1, and intra-daily variation of *α*1.

### Covariates

Demographics (sex, age, marital status) and socioeconomic status (level of education, employment) were included in the analysis as additional covariates (Farmer et al., 1988), as they are associated with psychopathology and sleep (Droomers, Schrijvers, & Mackenbach, 2001; Stamatakis, Kaplan, & Roberts, 2007). Additionally, we controlled for the mean activity level, which is known to correlate with depression and might contribute to observed group differences (Burton et al., 2013). For each period of interest, mean activity levels were estimated by averaging the total AC recorded by the actigraphy device over the period. Finally, we controlled for the frequency of zero AC, particularly significant during nighttime, that could influence the assessment of temporal correlations in activity fluctuations (Hu et al., 2001).

In the NESDA sample, antidepressant and benzodiazepine use was based on drug container inspection, with medications classified according to the World Health Organization Anatomical Therapeutic Chemical (ATC) coding system. Antidepressant and benzodiazepine use were considered present if participants reported usage exceeding 50% of the time (Difrancesco et al., 2019).

### Statistical analysis

We employed three statistical models to test our hypotheses.

#### Model A (between-subject comparison for hypothesis 1)

To determine differences in fractal activity patterns between the groups with different recency of depression diagnosis and those without depression history, we examined the data of the four NESDA groups (i.e. never-depressed group, current, recent, and remitted depression groups). We employed mixed ANOVA models in which fractal metrics (weekly *α*1, weekly *α*2, weekly Δ*α*, 4 h *α*1, and intra-daily variation in 4 h *α*1) served as outcomes, the group as a fixed factor, and subject ID as a random intercept. Adjustments were made for sex, age, education, mean activity level, and the number of points with zero value representing sleep/inactivity periods. Post-hoc analyses were performed to determine the differences between each pair of groups. In the models for 4 h *α*1, sleep/wake state and its interaction with the group were included as fixed factors, with post-hoc analyses conducted to explore group differences during sleep, wakefulness, and the sleep–wake transitions.

#### Model B (within-subject comparison for hypothesis 1)

To examine within-subject alterations in fractal activity patterns during the onset of a new depressive episode, we analyzed data from remitted individuals in the TRANS-ID study who experienced depressive symptom recurrence after antidepressant tapering. Data were categorized into three stages: before, during, and after the onset of depressive symptom recurrence. Mixed ANOVA models were performed, with fractal metrics as outcomes, depressive symptom recurrence stage as a fixed factor, and subject ID as a random intercept. Sleep/wake state effects and their interaction with the group were considered in models for 4 h *α*1. Adjustments were made for sex, age, education, mean activity level, and the number of points with zero value, with post-hoc analyses conducted to compare differences between each pair of stages.

#### Model C (between-subject comparison for hypothesis 2)

To determine whether euthymic fractal activity patterns predict the development of subsequent depressive symptoms with antidepressant tapering (hypothesis 2), we used data from all remitted individuals in the TRANS-ID study. For participants who developed depressive symptoms, only data preceding symptom onset were included. Mixed ANOVA models were employed, treating fractal metrics as outcomes and group (with or without subsequent depressive symptoms) as a fixed factor, with subject ID as a random intercept. Sleep/wake state effects and their interaction with the group were considered in models for 4 h *α*1. Adjustments were made for sex, age, education, mean activity level, and the number of points with zero value. Post-hoc analyses were conducted to compare recurrence status differences during sleep and wakeful periods separately.

All statistical analyses were performed using JMP (Version 16, SAS Institute Inc.), with statistical significance set at *p* < 0.05.

## Results

### Descriptive statistics of NESDA and TRANS-ID samples

Table 1a presents the demographic and clinical characteristics of NESDA participants. Among the 327 individuals, the mean age was 50.4 years (range 28–72), with 64.0% being female. No significant differences were observed in sex, marital status, or benzodiazepine usage between depression groups and controls. However, depressed individuals were more frequently unemployed, had lower education levels, higher mean depressive symptom scores, and a higher prevalence of psychotropic medication usage compared to controls. Participants in the recent depression group were younger compared to other groups. Assessment of depressive symptom severity and psychotropic medication usage was conducted up to 31 days prior to the actigraphy assessment.

**Table 1.** NESDA (a) and TRANS-ID (b) sample characteristic

(a) NESDA	Current depression *N* = 43	Recent depression *N* = 47	Remitted depression *N* = 151	Never-depressed *N* = 86	*p* value
Sex, female: *n* (%)^a^	29 (67.44)	32 (68.09)	100 (65.79)	49 (56.98)	0.465
Age, years: median (IQR)^b^	52 (15)	44 (18)	50 (23)	54 (22)	**0.039**
Employment, yes: *n* (%)^a^	15 (34.10)	21 (44.69)	97 (63.82)	47 (75.81)	**<0.001**
Education, university: *n* (%)^a^	13 (30.23)	20 (42.55)	66 (43.42)	53 (61.63)	**0.005**
Marital status, married: *n* (%)^a^	22 (51.16)	14 (29.79)	77 (50.66)	35 (56.45)	0.061
IDS, score: median (IQR)^b^	35 (13)	15 (9)	12 (12)	5 (4)	**<0.001**
Depression diagnosis present, assessment waves: mean (s.d.)^c^	4.15 (1.00)	3.56 (1.31)	1.77 (0.93)	–	**<0.001**
Psychotropic medication, frequent, any: *n* (%)^a^	28 (65.12)	26 (55.32)	47 (30.92)	4 (4.65)	**<0.001**
Antidepressants: *n* (%)^a^	21 (48.84)^d^	20 (42.55)^d^	28 (18.54)	0 (0)	**<0.001**^e^
Benzodiazepines: *n* (%)^a^	3 (6.98)	2 (4.26)	8 (5.30)	0 (0)	0.153

(b) TRANS-ID	Recurrence group *N* = 31	No recurrence group *N* = 19	*p* value
Sex, female: *n* (%)^a^	26 (83.87)	15 (78.95)	0.562
Age, years: median (IQR)^f^	49 (35)	46 (19)	0.220
Employment, yes: *n* (%)^a^	24 (77.42)	18 (94.74)	0.476
Education, university: *n* (%)^a^	27 (87.10)	14 (73.68)	0.095
Relationship, yes: *n* (%)^a^	25 (80.65)	15 (78.95)	0.628
SCL-90, all weeks, score: median (IQR)^f^	28 (12)	22 (10)	**<0.001**
SCL-90, week 1, score: median (IQR)^f^	23 (11)	25 (10)	0.699
Symptom recurrence after antidepressants tapering, days: mean (min to max)	43.75 (−50^g^ to 147)	–	–

IQR, interquartile range; IDS, Inventory of Depressive Symptomatology; SCL-90, Symptom Checklist.

a χ^2^ test.

b Kruskal–Wallis test.

c ANOVA.

d There was no significant difference between the current and recent depression groups (*p* > 0.05).

e Comparison was done between the depression groups excluding the control group.

f Wilcoxon test.

g Negative value means the lowest dose of antidepressants was taken after the recurrence of depressive symptoms.

Bold values indicate significant results (*p* < 0.05).

Table 1b outlines the demographic and clinical characteristics of TRANS-ID participants. Among the participants, 42 (84%) were employed, 41 (82%) had a university education, and 40 (80%) were married or in a relationship. No significant differences were noted in age, sex, education, relationship status, employment, or baseline SCL-90 score between individuals with and without depressive symptom recurrence. However, individuals experiencing depressive symptom recurrence exhibited significantly higher SCL-90 scores averaged across all weeks, compared to those without recurrence. The average time to symptom recurrence after reaching the lowest antidepressant tapering dose was 44 days, ranging from 50 days before to 147 days after reaching this dose. Notably, some participants experienced symptom recurrence during tapering while still on a higher antidepressant dose, achieving the lowest dose later in the tapering process.

### Differences between individuals according to depression recency (model A)

In NESDA participants, *weekly* mean *α*1 and *α*2 of all the groups approached 1 (current depression: *α*1 = 1.039 ± 0.010, *α*2 = 0.888 ± 0.017; recent depression: *α*1 = 1.055 ± 0.010, *α*2 = 0.851 ± 0.017; remitted depression: *α*1 = 1.042 ± 0.007, *α*2 = 0.851 ± 0.013; controls: *α*1 = 1.042 ± 0.009, *α*2 = 0.855 ± 0.015), indicating complex activity fluctuations with positive temporal correlations. After adjusting for covariates, no significant overall group differences were observed in weekly *α*1, *α*2, and Δ*α* (*p* > 0.16). However, the current depression group exhibited larger weekly *α*2 (0.888 ± 0.017) compared to the remitted depression group (0.851 ± 0.013, *post-hoc p* = 0.032). The recent depression group had a significantly larger Δ*α* (0.204 ± 0.022) than the current depression group (0.149 ± 0.022, *post-hoc p* = 0.041).

**Figure 1.**
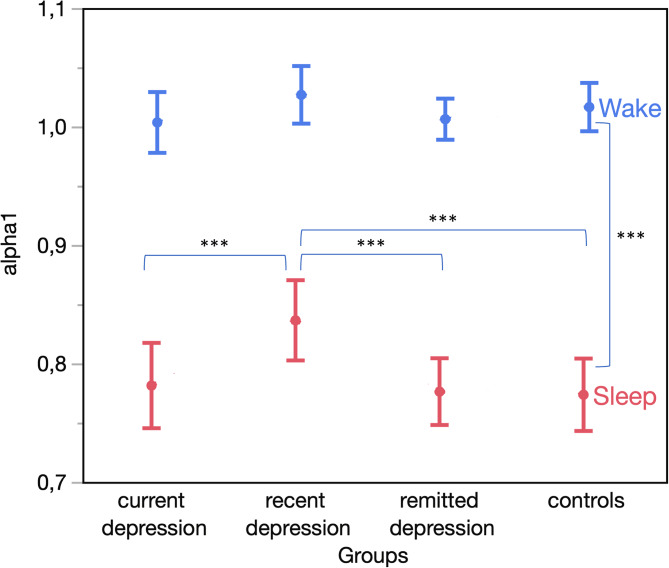
Sleep–wake difference in *α*1 for never-depressed and depression groups in the NESDA sample. *Note*: **p < 0.01; ***p < 0.001. The vertical comparison only shows the main effect of wake *v*. sleep.

Analysis of *4* *h* windows showed that *α*1 was larger during wakefulness (1.013 ± 0.008, suggesting a stronger temporal correlation) than during sleep (0.792 ± 0.013, suggesting more random fluctuations) (*p* < 0.001) with variations across groups (*p* = 0.080 for the interaction of group and sleep/wake) (Fig. 1). Specifically, the recent depression group showed higher *α*1 during sleep (0.836 ± 0.017) compared to controls (0.773 ± 0.016, *post-hoc p* < 0.001), remitted depression (0.776 ± 0.014, *post-hoc p* < 0.001), and current depression groups (0.781 ± 0.018, *post-hoc p* = 0.006). No significant group difference in *α*1 was observed during wakefulness and in intra-daily variation of *α*1 (*p* > 0.1).

As a sensitivity analysis, we investigated the potential influence of employment and marital status on observed group differences. While employment was associated with lower *α*1 (*p* = 0.001), controlling for both factors did not alter group differences in sleep *α*1. These differences also persisted after adjusting for education.

### Within-subject changes during depressive symptom recurrence (model B)

Among TRANS-ID participants with depressive symptom recurrence, no significant differences were observed in *weekly α*1, *α*2, or Δ*α* across different periods (before, during, and after symptom onset) (*p* > 0.1).

**Figure 2.**
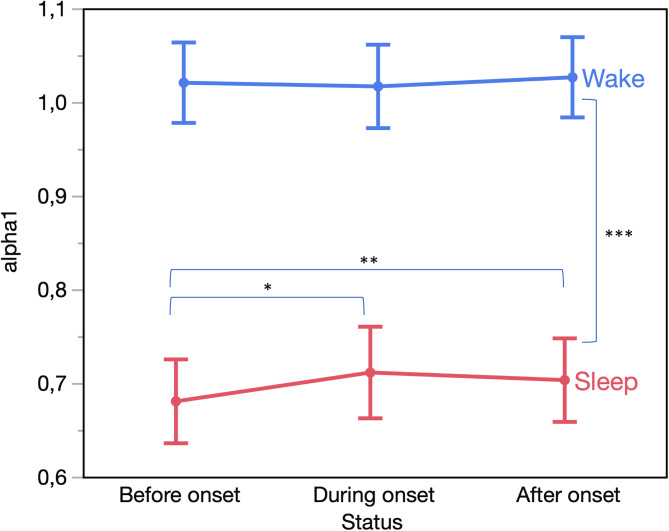
Sleep–wake difference in *α*1 for the remitted individuals with a clinically relevant recurrence of depressive symptoms in the TRANS-ID sample. *Note*: *p < 0.05; **p < 0.01; ***p < 0.001.

Analysis of *4* *h* windows revealed a sleep–wake difference in *α*1 consistent with that found in NESDA, i.e. *α*1 during sleep (0.698 ± 0.022) was smaller than during wakefulness (1.021 ± 0.021, *p* < 0.001). We found a significant interaction between the sleep–wake difference and symptom recurrence status (*p* = 0.017) (Fig. 2) with sleep *α*1 increasing during the week of symptom onset from 0.680 ± 0.022 before the onset to 0.711 ± 0.025 (*post-hoc p* = 0.011) during the week of onset and remaining elevated thereafter (0.703 ± 0.022, *post-hoc p* < 0.001), with no significant changes during wakefulness (*p* > 0.1). Additionally, intra-daily variation of *α*1 was higher before symptom onset (0.178 ± 0.012) compared to the week of onset (0.169 ± 0.012, *post-hoc p* = 0.044).

### Differences in euthymic fractal activity patterns between remitted participants with and without subsequent depressive symptom recurrence (model C)

**Figure 3.**
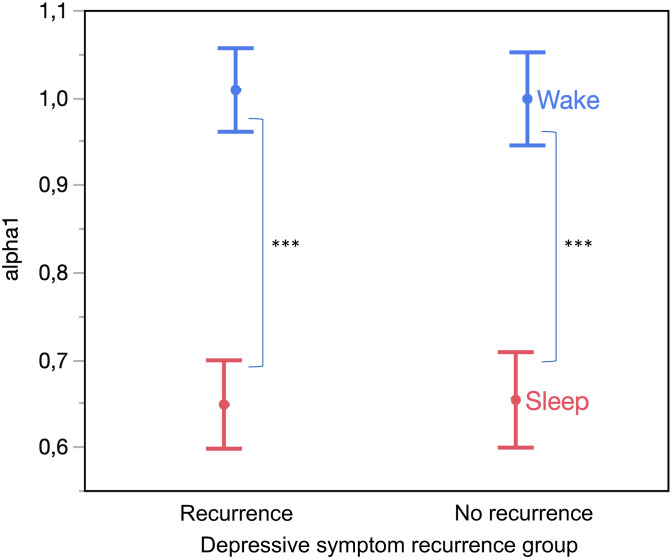
Sleep–wake difference in *α*1 for the remitted individuals before a recurrence of depressive symptoms in the TRANS-ID sample. *Note*: ***p < 0.001.

Among TRANS-ID participants, those who later developed a recurrence of depressive symptoms did not show significant differences in fractal metrics before the onset of these symptoms when compared to participants who did not experience any recurrence (*p* > 0.1, Fig. 3 shows *α*1 only).

## Discussion

This study aimed to explore fractal patterns in motor activity fluctuations among individuals with different recency of depression diagnoses during both sleep and wakefulness. Our findings revealed distinct alterations in fractal activity fluctuations during sleep among participants with recent depression diagnoses (within 1–6 months before actigraphy assessment) compared to never-depressed, current depression, and remitted depression individuals. We also observed changes in fractal activity patterns during sleep among remitted participants experiencing a recurrence of depressive symptoms. However, we did not find differences in euthymic fractal activity patterns between remitted individuals with and without subsequent recurrence of depressive symptoms. These results suggest that changes in fractal activity patterns during sleep may occur parallel to or following the onset of depressive symptom recurrence, indicating a complex interplay between motor activity, sleep–wake patterns, and depression dynamics.

For the first time, we identified a sleep–wake difference in fractal motor activity fluctuations among individuals diagnosed with depression. Notably, Aybek et al. (2012) identified differences in fractal motor activity patterns in individuals with depression; however, our study further showed the changes in fractal patterns across different stages of depressive episodes and across sleep and wakefulness. While one might expect reduced activity correlations or more random fluctuations during sleep due to suppressed motor control, we found stronger temporal correlations during sleep in individuals with recent depression and remitted individuals experiencing depressive symptom recurrence. This suggests more ‘wake-like’ activity patterns during sleep in these individuals. Interestingly, this phenomenon could not simply reflect increased nighttime awakenings as commonly reported in individuals with major depression (Medina, Lechuga, Escandón, & Moctezuma, 2014), as our models were adjusted for mean activity levels and inactivity duration during sleep. Furthermore, the alterations did not seem related to transitional periods during the sleep–wake cycle, as data around sleep onset and waking up were excluded from the analysis.

The benefits of fractal regulation of motor activity may lie in its role in promoting adaptability and resilience. Complex systems, like the human body, thrive on variability and flexibility to respond to internal and external challenges effectively (Pincus & Metten, 2010). Fractal motor activity patterns provide a balance between randomness and regularity, allowing for effective adjustments to changing conditions. The observed alterations in fractal activity patterns during sleep may reflect adjustments in this balance based on physiological and psychological conditions. Notably, the ‘tilted balance’ during sleep in controls did not lead to total randomness in motor activity fluctuations, indicating partially active motor control during sleep or the active dynamic control of sleep–wake stage transitions. The ‘wake-like’ activity patterns observed during sleep in depression participants may offer new insights into the effect of depression on sleep dynamics and could be valuable for clinical assessment of depression progression.

Intriguingly, altered fractal activity patterns during sleep were observed specifically in individuals with recent (1–6 months) but not current depression diagnoses (within 1 month). This unexpected finding may be attributed to uncontrolled factors influencing motor activity regulation, such as changes in food intake or exercise, which could differ between groups (Li et al., 2017a). Additionally, this discrepancy might be a chance finding or indicative of a delayed effect of depression. Future studies are needed to confirm these observations. Notably, previous research on the same dataset reported differences in physical activity levels among different recency groups (Minaeva et al., 2020), suggesting that fractal activity patterns and physical activity levels offer complementary information about motor activity regulation during sleep.

Another notable observation was that the group with current depression appeared to have more ‘favorable’ fractal activity patterns across two time-scale regions (i.e. overall less disrupted fractal activity patterns) than the group with recent depression. These differences could not be attributed to variations in antidepressant or benzodiazepine treatment frequency or dosage or the chronicity of depression diagnosis. It is plausible that the altered fractal patterns in the recent depression group reflect a ‘recovery phase’ characterized by energy conservation and altered sleep structure after a depressive episode (Borbély & Achermann, 1992; Webb, 1988). However, further research, preferably utilizing within-subject designs, is needed to confirm these interpretations.

Similarly, the changes in fractal activity patterns during and after the onset of depressive symptom recurrence in remitted participants align with the hypothesized ‘delayed’ effect of depression. This is further supported by the absence of differences in euthymic fractal patterns between individuals with and without subsequent depressive symptoms. This dysregulation in motor system dynamics during sleep may signify an allostatic overload associated with previous depressive episodes (McEwen & Karatsoreos, 2015), leading to the re-emergence of depressive symptoms. The findings underscore the need for comprehensive assessments of motor activity dynamics in understanding depression progression and recurrence.

Despite its strengths, including large sample size and long-term actigraphy assessments, and both cross-sectional design and within-subject design, our study has limitations. TRANS-ID participants were not entirely symptom-free at baseline, potentially affecting the detection of differences in fractal patterns between different periods. Additionally, the TRANS-ID study, primarily designed for detecting within-person changes, had a relatively small sample size for between-subject comparisons. Moreover, considering the heterogeneity of depression, the recent and current depression groups may represent distinct subtypes, each with unique underlying neurobiological processes (Schrijvers, Hulstijn, & Sabbe, 2008). Alterations in fractal patterns in motor activity might be more closely linked to specific depression subtypes, such as melancholic or atypical depression (Tonon et al., 2017). Although our study found no differences between the recent and current depression groups based on melancholic or atypical IDS scales in NESDA, it is important to note that these scales provide only an approximation and may not be precise measures for identifying specific depression subtypes. Furthermore, differences in actigraphy devices and algorithms between the NESDA and TRANS-ID datasets prevented direct comparisons, even though we were able to observe a similar pattern. Finally, all calculations were conducted post-data gathering, meaning real-time data collection and processing was not possible. Future research addressing these limitations is warranted to further elucidate the mechanisms underlying changes in fractal activity patterns associated with depression.

In conclusion, advances in wearable technology opened up new possibilities for continuous and unobtrusive monitoring of physiological signals, such as motor activity, over a longer period. Harvesting information about depressive symptoms and their recurrence during normal daily life via smartphones and wearable devices could potentially provide low-cost, low-burden, yet powerful tools, potentially facilitating remote medicine in individuals with depression. Fractal activity patterns may provide unique insights into the relationship between depression and motor activity control, independent of traditional behavioral measures. Incorporating fractal analysis into clinical practice could facilitate timely diagnosis and treatment of depression, ultimately improving patient outcomes.

## Supporting information

Minaeva et al. supplementary material 1 Minaeva et al. supplementary material

Minaeva et al. supplementary material 2 Minaeva et al. supplementary material

Minaeva et al. supplementary material 3 Minaeva et al. supplementary material

## Data Availability

Data are not freely available in a public repository because of restrictions related to data containing information that could compromise the participants' privacy. However, collaboration on the NESDA data study is possible and can be requested via publication plans for which information is provided on www.nesda.nl. Collaboration on the Transitions in Depression (TRANS-ID) Tapering study data is possible and can be requested viah.riese@umcg.nl.

## Supplementary material

The supplementary material for this article can be found at https://doi.org/10.1017/S0033291724002769.
