# Supplementary material for: Fractal motor activity during wakefulness and sleep: a window into depression recency and symptom recurrence
Source: Psychol Med. 2024 Dec 2;54(15):4429–37. doi: 10.1017/S0033291724002769 (PMC11650180; doi:10.1017/S0033291724002769)
Supplement: Minaeva et al. supplementary material 1 — Minaeva et al. supplementary material [file S0033291724002769sup001.docx]

**Supplementary Material 1. Flowcharts of inclusion process for the NESDA and TRANS-ID datasets**


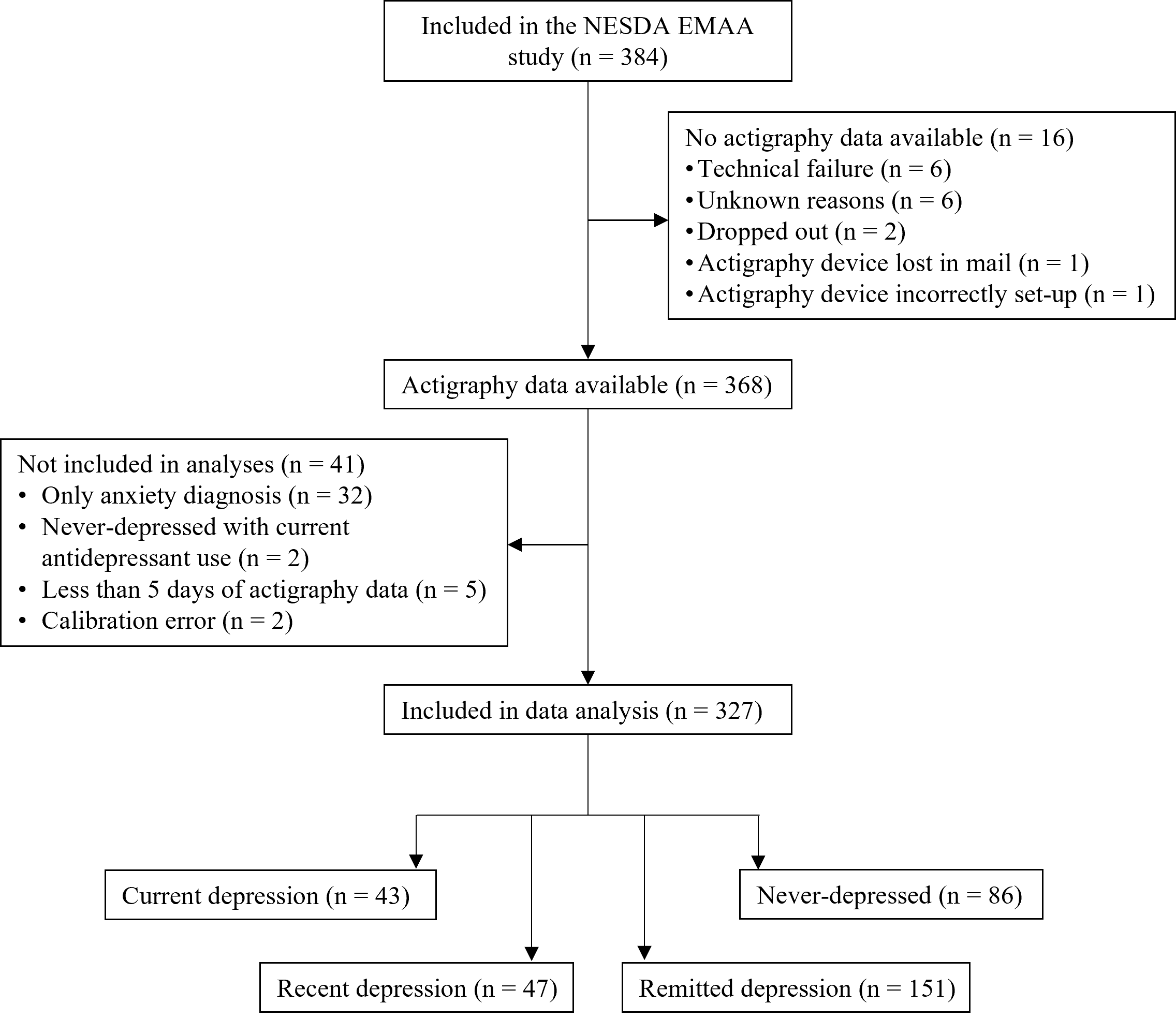


**Figure 1a. Flowchart of the NESDA participants**


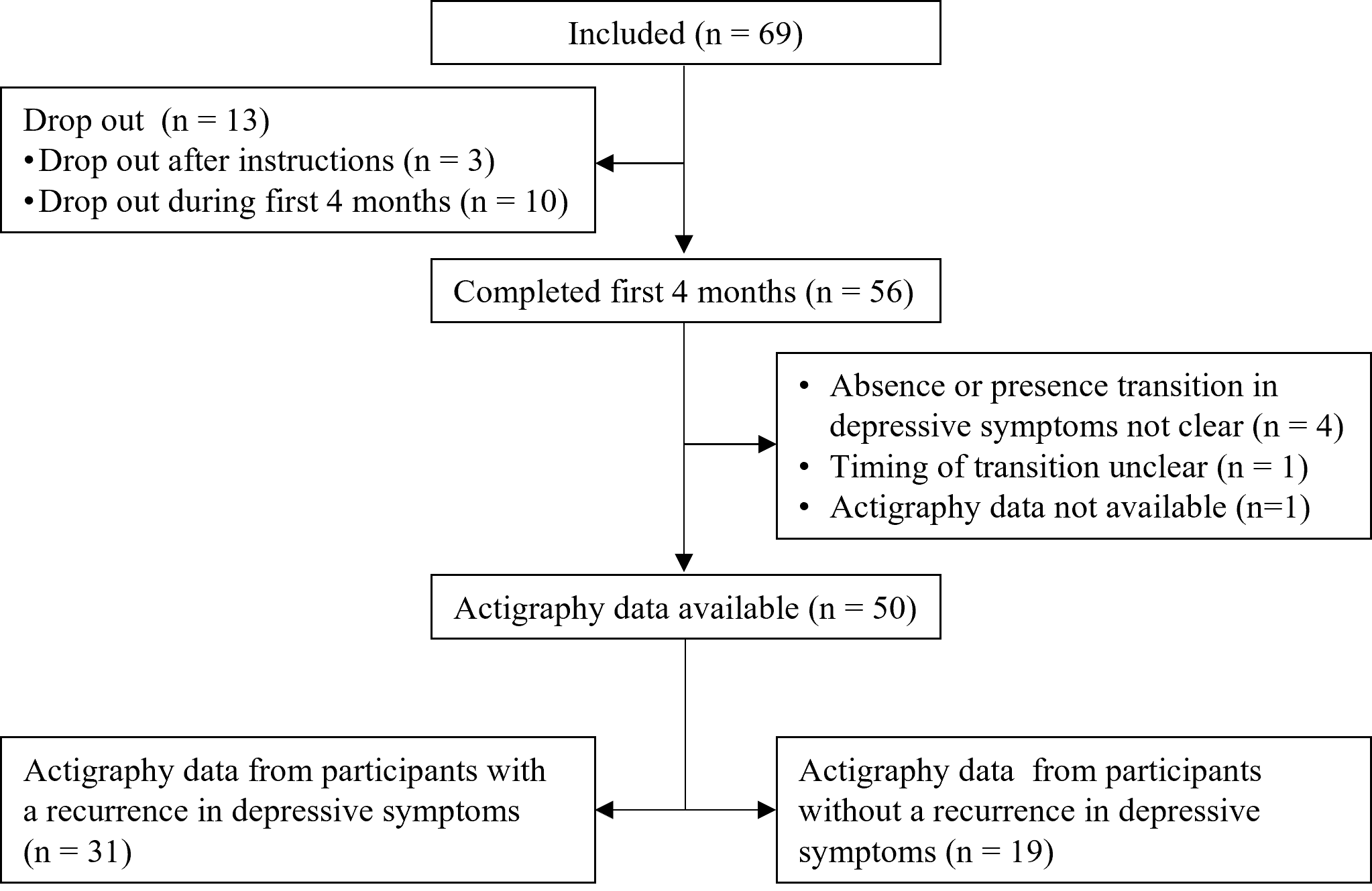


**Figure 1b. Flowchart of the TRANS-ID participants**
