# Supplementary material for: Fractal motor activity during wakefulness and sleep: a window into depression recency and symptom recurrence
Source: Psychol Med. 2024 Dec 2;54(15):4429–37. doi: 10.1017/S0033291724002769 (PMC11650180; doi:10.1017/S0033291724002769)
Supplement: Minaeva et al. supplementary material 2 — Minaeva et al. supplementary material [file S0033291724002769sup002.docx]

**Supplementary Material 2. Data preprocessing steps**

**TRANS-ID data**

1. Plot the data in Matlab (csv files generated by MotionWare) to check for visible abnormalities (use ezActi);
2. Equalize all files to start at 00:00 (to be able to analyze the daily rhythm);
3. Identify and mark gaps using ezActi;
4. Run two sets of DFA:
   1. On a 7-day window (168 hours) – two scales: small (5-90 min) and large (120-480 min);
   2. On a 4-hour window – one scale 5-40 min;

Alpha should have a normal distribution from 0.5 to 1.5

*Checking data quality*

- Check the distribution of goodness for daytime and nighttime
  - For daytime, goodness should be > 0.8
  - For nighttime – check the distribution; goodness should be > 0,4
- Restrict maxscale to at least 20 min or more

**NESDA data**

1. **Perform recalibration and computation of activity counts ourselves**
   1. Use R package “GENEAread” to read encrypted .bin files and recalibrate them -> recalibrated .bin files are created;
   2. Save recalibration output in a text file (“**Recalibration summary NESDA**”) and pay attention to the status (should be no errors), scale correction (should be close to 0), calibration error after, and offset correction (should be less than 0.005 – the threshold for the noise we chose based on analyzed stationary period);
   3. Transform recalibrated .bin files to xyz-files for Matlab (saving as csv takes 4,5 Gb per file);
   4. Use Matlab script to test recalibration (**testing.m**); first, plot the data and find a single stationery segment -> find a threshold for x, y, and z (for noise) and sampling frequency;
   5. Use Matlab script for stationary segments (**stationaryseg.m**). Using sampling frequency, the threshold for minimal increment for x,y, and z, and epoch length (60 sec), analyze xyz-data files and identify all stationary segments -> plot the data highlighting the stationary segments -> plot the data on the 3D sphere to see the distribution of the data points on the sphere (calibrated data points should be on the surface, not inside the sphere or away from the surface) -> if plots look good and the data calibration is sufficient then move to create activity counts (AC);
   6. Use Matlab script (**gen_act.m**) to compute AC from the xyz-files; plots will be created as well;
   7. Due to the lack of space on the server, we first created a Matlab script (**compressmatfile.m**) for compressing the xyz-files by removing unnecessary data (temperature, light, button, and datastamps except the first one to make sure each file starts at the same time);
   8. To speed up the process, we updated the script **gen_act.m** to **gen_act2.m** to be able to produce AC from compressed xyz-files.
2. Make all NESDA AC files start at 00:00 instead of 19:00;
3. Assess data quality by plotting the data of each individual in ezActi;
4. Identify and mark the gaps
5. Checking data quality and excluding data points that don’t fulfill the inclusion criteria

- Check the distribution of goodness for daytime and nighttime
  - For daytime, goodness should be > 0.8
  - For nighttime – check the distribution; goodness should be > 0,4
- Restrict maxscale to at least 20 min or more

*Finding agreement between habitual sleep schedule and actigraphy-estimated sleep*

1. Turn habitual sleep variables to a numeric format from the time format.
2. Create variables that represent the agreement between habitual and actigraphy sleep times (for sleep onset, sleep offset, and TIB).
3. If the difference is less than 2 hours (+/- 1 hour), then consider it as an acceptable agreement, if more than 2 hours, then no agreement, and if one or both components are missing, then the agreement is unclear.
   1. ***might require a manual check for all unclear agreements**
4. Repeat calculating the agreement for all three sleep variables.
