# Supplementary material for: Fractal motor activity during wakefulness and sleep: a window into depression recency and symptom recurrence
Source: Psychol Med. 2024 Dec 2;54(15):4429–37. doi: 10.1017/S0033291724002769 (PMC11650180; doi:10.1017/S0033291724002769)
Supplement: Minaeva et al. supplementary material 3 — Minaeva et al. supplementary material [file S0033291724002769sup003.docx]

**Supplementary Material 3. Matlab scripts for data preprocessing**

1. **testing.m**

clear all;

%filename = '100001.mat';

%filename = '100001_xyz_calibrated.mat';

[filename,infolder] = uigetfile();

A1=load(fullfile(infolder,filename));

n=length(A1.A.x);

epoch = max(diff(A1.A.timestamp));

ix=[1:n];

A1.A.timestamp=A1.A.timestamp/3600/24;%day **in** unit

t1=A1.A.timestamp(1);

t0str='16-Nov-2014 19:00:00';

t0=datenum(t0str);

tcorrection = t0-t1;

A1.A.timestamp = A1.A.timestamp + tcorrection;

figure();

subplot(3,1,1)

plot(ix,A1.A.x);

subplot(3,1,2)

plot(ix,A1.A.y);

subplot(3,1,3)

plot(ix,A1.A.z);

**return**;

%select the segment without movement

tn=400000;

% jjj=[1:400000];%eyeball the seg%**for** 1000xx

jjj=[1:400000];%eyeball the seg%**for** 1000xx

xlim([1,tn]);

testx=A1.A.x(jjj);

testy=A1.A.y(jjj);

testz=A1.A.z(jjj);

deltax=diff(testx);

deltay=diff(testy);

deltaz=diff(testz);

figure();

subplot(3,1,1)

plot([1:tn-1],deltax);

subplot(3,1,2)

plot([1:tn-1],deltay);

subplot(3,1,3)

plot([1:tn-1],deltaz);

figure();

subplot(3,1,1)

hist(deltax,50);

subplot(3,1,2)

hist(deltay,50);

subplot(3,1,3)

hist(deltaz,50);

values=unique(deltax);

format long

resol=max(abs(diff(values)))%resolution of the sensor

%threshold **for** no change **in** acceleration (**in** each direction)

threshx = max(abs(deltax))

threshy = max(abs(deltay))

threshz = max(abs(deltaz))

va=testx.*testx+testy.*testy+testz.*testz;

%3-d plot of acceleration vector

[x,y,z]=sphere(50);

figure();

h=surf(x,y,z);

set(h, 'FaceAlpha', 0.5)

shading interp;

get(gcf,'Renderer');

hold on

scatter3(testx,testy,testz,'k');

get(gcf,'Renderer');

xlabel('x');ylabel('y');zlabel('z');

legend({'Unit sphere','Before'});

%legend({'Unit sphere','Before','After'});

1. **stationaryseg.m**

*%find the stationary segments*

clear all

sf = 30;*%sampling frequency*

thresholdincr = 0.05;*%threshold for minimal increment (otherwise replace the value with 0)*

threshold = 300*sf;*% minimal duration for segments without movement*

[filename,infolder] = uigetfile();

*%filename = '100001_xyz_calibrated.mat';*

*% filename = '100001.mat';*

A1=load(fullfile(infolder,filename));

*% return;*

n=length(A1.A.x);

epoch = max(diff(A1.A.timestamp));

ix=[1:n];

dx = diff(A1.A.x);

dy = diff(A1.A.y);

dz = diff(A1.A.z);

*%% find those points with increments < threshold*

ii = find(abs(dx)<thresholdincr & abs(dy)<thresholdincr & abs(dz)<thresholdincr);

if isempty(ii)

stationaryData.Messages = [stationaryData.Messages,'Error! No points with change < noise level!'\n];

**return**;

end

jj=find(diff(ii)>1);

starti=[];

endi=[];

slen=0;

*% starti(1)=ii(1);*

*% for i=1:length(jj)*

*% endi(i)=ii(jj(i));*

*% starti(i+1)=ii(jj(i)+1);*

*% end*

*% endi(length(jj)+1)=ii(end);*

starti = [ii(1);ii(jj+1)];

endi = [ii(jj);ii(**end**)];

duration = endi-starti+1;

*%find the segments with duration > threshold*

kk=find(duration>threshold);

if isempty(kk)

stationaryData.Messages = [stationaryData.Messages,'Error! No segments longer than ',num2str(threshold),' points!\n'];

**return**;

end

duration = duration(kk);

starti = starti(kk); endi = endi(kk);

*%plot the data and highlight those segments identified*

len = size(dx,1);

selt=[1:len]';

x=nan(len+1,1);

y=nan(len+1,1);

z=nan(len+1,1);

seldx = nan(len,1);

seldy = nan(len,1);

seldz = nan(len,1);

for i=1:length(duration)

seldx(starti(i):endi(i))=dx(starti(i):endi(i));

seldy(starti(i):endi(i))=dy(starti(i):endi(i));

seldz(starti(i):endi(i))=dz(starti(i):endi(i));

x(starti(i):endi(i))=A1.A.x(starti(i):endi(i));

y(starti(i):endi(i))=A1.A.y(starti(i):endi(i));

z(starti(i):endi(i))=A1.A.z(starti(i):endi(i));

end

f1=figure();

subplot(3,1,1)

plot([1:len],dx,'b');hold on; plot(selt,seldx,'r');

subplot(3,1,2)

plot([1:len],dy,'b');hold on; plot(selt,seldy,'r');

subplot(3,1,3)

plot([1:len],dz,'b');hold on; plot(selt,seldz,'r');

outfile1 = [filename(1:**end**-4),'_statseg.fig'];

saveas(gcf,fullfile(infolder,outfile1));

*%3-d plot of acceleration vector*

[x0,y0,z0]=sphere(50);

f2=figure();

h=surf(x0,y0,z0);

set(h, 'FaceAlpha', 0.5)

shading interp;

get(gcf,'Renderer');

hold on

scatter3(x,y,z,'r');

get(gcf,'Renderer');

xlabel('x');ylabel('y');zlabel('z');

legend({'Unit sphere','Before'});

outfile2 = [filename(1:**end**-4),'_sphere.fig'];

saveas(gcf,fullfile(infolder,outfile2));

*%legend({'Unit sphere','Before','After'});*

1. **gen_act2.m**

*%%load calibrated accelerometer raw data files in a folder and generate*

*%%activity counts for each file*

clear all

*%%Parameters*

sf=30;*%sampling frequency in Hz*

thresholdincr = 0.05;*%threshold for minimal increment (otherwise replace the value with 0)*

epoch = 60;*%epoch length (60 seconds; 15 seconds for longer range of time scales)*

pinepoch = epoch*sf; *% number of points in an epoch*

*%threshold2 (optional)*

*%% choose a folder where all the calibrated data files are kept*

infolder = uigetdir()

filetype = '*cal*.mat';

filelist = dir(fullfile(infolder,filetype));

newfolder = 'Activity';*%a new subfolder within the selected folder where all activity count files will be saved*

mkdir(infolder,newfolder);

*%find the correction for timestamp*

t00='01-Jan-1970 00:00:00';

tcorr = datenum(t00);

*% t0str = '16-Nov-2014 19:00:00';*

*% t1str = '14-Nov-0044 19:00:00';*

*% tcorr = datenum(t0str)-datenum(t1str);*

*%for each file, load the data and calculate activity counts*

len = size(filelist,1);

for i=1:len

filename = filelist(i).name;

disp(['File ',num2str(i),': ',filename]);

A1=load(fullfile(infolder,filename));

dx = abs(diff(A1.x));

dy = abs(diff(A1.y));

dz = abs(diff(A1.z));

dx = floor(dx/thresholdincr);

dy = floor(dy/thresholdincr);

dz = floor(dz/thresholdincr);

rawa= sqrt(dx.*dx + dy.*dy + dz.*dz);

nac = floor(length(dx)/pinepoch);

reallen= nac*pinepoch;

act=sum(reshape(rawa(1:reallen),pinepoch,nac),1)*thresholdincr;*%*

*%starttime*

startt = A1.timestamp(1)/86400 + tcorr;

timestr = datestr(startt,30);*%'yyyymmddTHHMMSS'*

outfile = [filename(1:6),'_',timestr,'_',num2str(epoch),'.act'];

fid=fopen(fullfile(infolder,newfolder,outfile),'w');

fprintf(fid,'%.4f\n',act);

fclose(fid);

*%plot data and save the figure*

h = figure();

plot([0:nac-1]*epoch/3600,act);

xlabel('Time (h)');ylabel('Activity');

title(['Participant: ', filename(1:6),' StartTime: ',timestr]);

xticks([0:24:(nac-1)*epoch/3600]);

ax = gca; ax.XGrid = 'on';

outfile2 = [filename(1:6),'_',timestr,'_',num2str(epoch),'.fig'];

saveas(h,fullfile(infolder,newfolder,outfile2));

close(h);

end

1. **compressmatfile.m**

*%% For each .mat file in a folder, remove the data of timestamp, light, button, and temperature while keeping only the starttime, and x, y, z.*

*% [filename,infolder] = uigetfile();*

infolder = uigetdir()

filetype = '*cal*.mat';

filelist = dir(fullfile(infolder,filetype));

len = size(filelist,1);

for i=1:len

filename = filelist(i).name;

disp(['File ',num2str(i),': ',filename]);

A1=load(fullfile(infolder,filename));

A1.A.timestamp(2:**end**)=[];

A1.A.light=[];

A1.A.button=[];

A1.A.temperature=[];

C = A1.A;

save(fullfile(infolder,filename),'-struct','C');

end
